# Deletion of the BMP receptor BMPR1a impairs mammary tumor formation and metastasis

**DOI:** 10.18632/oncotarget.4413

**Published:** 2015-06-10

**Authors:** Michael W. Pickup, Laura D. Hover, Yan Guo, Agnieszka E. Gorska, Anna Chytil, Sergey V. Novitskiy, Harold L. Moses, Philip Owens

**Affiliations:** ^1^ Department of Surgery and Center for Bioengineering and Tissue Regeneration, University of California at San Francisco, San Francisco, CA, USA; ^2^ Department of Pathology, Microbiology and Immunology, Vanderbilt University Medical Center, Nashville, TN, USA; ^3^ Vanderbilt Ingram Cancer Center, Center for Quantitative Sciences, Nashville, TN, USA; ^4^ Department of Cancer Biology, Vanderbilt Ingram Cancer Center, Vanderbilt University, Nashville, TN, USA

**Keywords:** BMPR1a, BMP, breast cancer, EMT, metastasis

## Abstract

Bone Morphogenetic Proteins (BMPs) are secreted cytokines/growth factors belonging to the Transforming Growth Factor β (TGFβ) family. BMP ligands have been shown to be overexpressed in human breast cancers. Normal and cancerous breast tissue display active BMP signaling as indicated by phosphorylated Smads 1, 5 and 9. We combined mice expressing the MMTV.PyMT oncogene with mice having conditional knockout (cKO) of BMP receptor type 1a (BMPR1a) using whey acidic protein (WAP)-Cre and found this deletion resulted in delayed tumor onset and significantly extended survival. Immunofluorescence staining revealed that cKO tumors co-expressed Keratin 5 and mesenchymal cell markers such as Vimentin. This indicates that epithelial-to-mesenchymal (EMT)-like transitions occurred in cKO tumors. We performed microarray analysis on these tumors and found changes that support EMT-like changes. We established primary tumor cell lines and found that BMPR1a cKO had slower growth *in vitro* and *in vivo* upon implantation. cKO tumor cells had reduced migration *in vitro*. We analyzed human databases from TCGA and survival data from microarrays to confirm BMPR1a tumor promoting functions, and found that high BMPR1a gene expression correlates with decreased survival regardless of molecular breast cancer subtype. In conclusion, the data indicate that BMP signaling through BMPR1a functions as a tumor promoter.

## INTRODUCTION

The Bone Morphogenetic Protein (BMP) pathway is a critical regulator of development and belongs to the cytokine growth factor TGFβ family. Since their discovery in 1965, BMPs have been found to have roles in regulating cellular differentiation and progenitor self-renewal [1]. BMP ligands are secreted with prodomains, which must be cleaved and processed into active dimers that bind to type I and type II serine/threonine kinase receptors. Upon ligand binding and receptor oligomerization type I receptors phosphorylate Smads 1, 5 and 8(mouse)/9(human) that bind with Smad4 to shuttle to the nucleus where they bind site-specific DNA regulatory elements and regulate the transcription of target genes. Canonical BMP target genes in response to ligand stimulation are *ID1* as well as the inhibitory Smads 6 and 7, which function in a negative feedback manner thus tightly regulating BMP signaling [2-4].

BMP activity has largely been viewed as tumor suppressive as demonstrated by loss and gain of function of BMP signaling components. When BMPR2 is expressed as a dominant negative in a mouse model of breast cancer, it enhances tumor metastasis through a paracrine inflammatory microenvironment [5]. Interestingly, patients with germline mutations in BMPR1a develop Juvenile Polyposis Syndrome, which is characterized by the development of hamartomas and mice with targeted deletion of BMPR1a in skin develop similar hamartomatous lesions [6-10]. Treatment of most normal and cancerous cells with BMP ligands reduces cell proliferation and growth and, similar to TGFβ treatment, induces transcription of cyclin dependent kinases p21/27/57 to repress the MYC oncogene [11-13]. Treatment of cells with BMP ligand antagonists such as Noggin leads to increased cell proliferation and the BMP antagonist Coco promotes breast cancer metastasis [14, 15].

Contrary to established tumor suppressive roles, breast cancer cell migration and invasion is enhanced when cells are treated with BMP ligands [16, 17]. When BMP receptors are overexpressed in cells, they can also demonstrate tumor-promoting phenotypes such as increased invasion and metastasis [18]. Small molecule kinase antagonists to BMP receptors have also been shown to inhibit growth of tumors and their metastatic ability in breast, lung, and prostate cancer cells [19-21]. Additionally, when cells are treated with certain compositions of ligand heterodimers this can enhance their cancer stem cell ability [22]. Further experiments have demonstrated that BMP growth inhibition of cancer cells is actually promoting the dormant cancer stem cell fate [23]. Recently it has been shown that lung cancer cells resist chemotherapy by activating BMPR1a and that loss of BMPR1a sensitizes lung cancer cells to targeted chemotherapy [24].

With recent reports indicating conflicting results to BMP's role in tumor progression, it is important to determine whether BMP signaling is tumor promoting or tumor suppressive. Recent reviews highlighted these potential dual roles for BMPs in cancer [25, 26]. We have conditionally deleted BMPR1a in a breast cancer mouse model (Polyoma middle T–PyMT) to determine tumor suppressive or promoting functions. We found that loss of BMPR1a resulted in mammary tumors with EMT-like changes, but with delayed growth and progression.

## RESULTS

### BMPR1a deletion in mammary carcinomas delays tumor onset and progression

**Figure 1:**
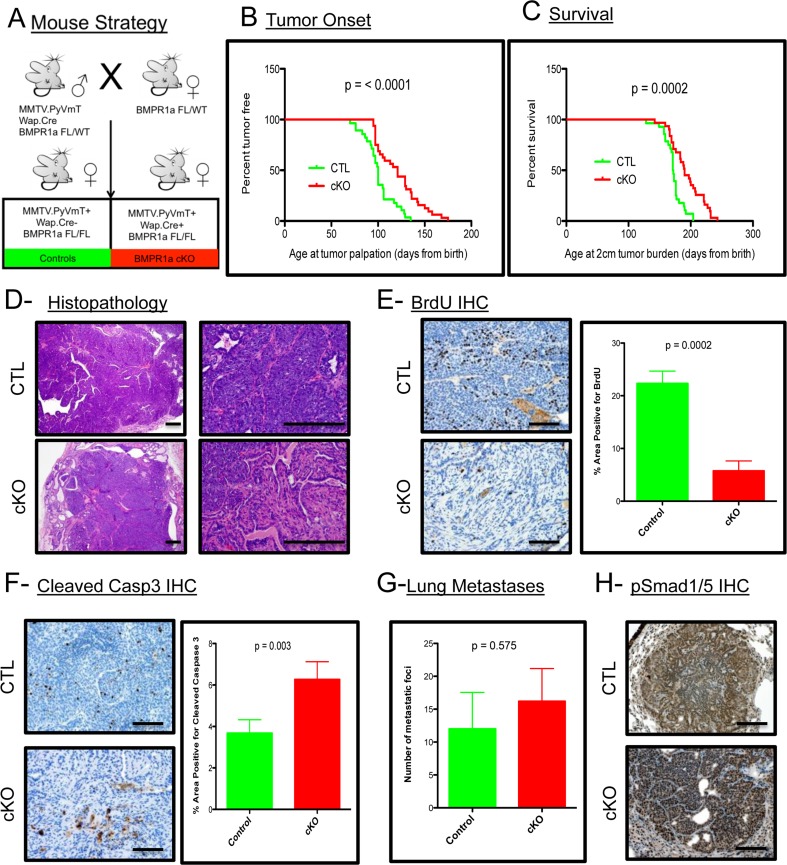
BMPR1a deletion in mammary carcinomas delays tumor onset and progression **A.** Breeding strategy of mice with males harboring Cre and oncogene PyMT. All mice have a heterozygous allele of Floxed BMPR1a. Control (CTL) mice (*n* = 31) lack Cre and conditional knockout (cKO) express Cre and are homozygous for BMPR1a floxed alleles (*n* = 29). **B.** Tumor onset was indicated by the day when tumors were first palpated from the day of birth. **C.** Survival was limited to mice bearing tumors of 2 cm of size in any direction and were euthanized and recorded as days since birth. **D.** Representative image of H&E Staining of primary spontaneous tumors at low and high magnification compared with cKO tumors. **E.** Representative image of IHC for BrdU incorporation and quantitation in primary tumors. **F.** Representative image of IHC for Cleaved-Caspase 3 and quantitation. **G.** Lung metastases were counted by wholemount Hematoxylin stain under a dissecting scope. **H.** Representative image of IHC for phospho-Smad 1/5 was performed on lung metastases. Error bars indicate SEM. Scale bars indicate 200μm.

To address the contribution of BMP signaling in the mammary epithelium to the promotion and progression of mammary carcinomas, we utilized the established PyMT mouse model [27]. This model was crossed with a Whey Acidic Protein (WAP) Cre mouse [28] to induce Cre mediated recombination and loss of the BMP receptor type 1a (BMPR1a) in mice harboring floxed alleles [29] (Figure 1A). The initiation of tumorigenesis and progression of the tumors to 2 cm are significantly delayed upon loss of BMP signaling (Figure 1B and 1C). Histological analysis of the resulting tumors shows a similar carcinoma appearance typical with this oncogene in the C57BL/6 strain (Figure 1D). Additionally, the resulting cKO tumors displayed pathological features not present in the control tumors, such as focal regions of desmoplasia and squamous cell carcinoma (SCC)-like morphology as evidenced by keratin pearls (Suppl. Figure 1A). BrdU staining indicated a significant decrease in proliferation in cKO tumor epithelium (Figure 1E). There was also a significant increase in cell death as indicated by staining for cleaved-Caspase 3 (Figure 1F). Immunohistochemistry for phospho-Smad1/5 shows the phenotypic changes are complemented with inhibition of BMP signaling in the tumor epithelium (Suppl. Figure 1B). Wap.Cre was chosen to target the mammary gland to avoid potential developmental defects and indeed no Cre expression (GFP+ Cells) could be detected in developing mammary glands (Suppl. Figure 1C). However, tumors displayed mosaic expression of GFP+ cells indicating recombination that could be focal and heterogeneous (Suppl. Figure 1D). Interestingly, none of the lung metastases that formed from cKO tumors contained GFP+ cells, which suggested that only cells that had intact BMPR1a were capable of establishing lung metastases (Suppl. Figure 1E). All metastatic lesions formed were positive for phospho-Smad1/5, indicating active BMP signaling in the metastasized cells (Figure 1H). Despite changes in primary tumors, no significant difference in the number of lung metastases was observed (Figure 1G). This data indicates that the loss of BMP signaling in the mammary epithelium significantly decreases the tumorigenic potential of the PyMT-induced mammary tumors.

### BMPR1a deletion in mammary carcinomas results in EMT-like changes

**Figure 2:**
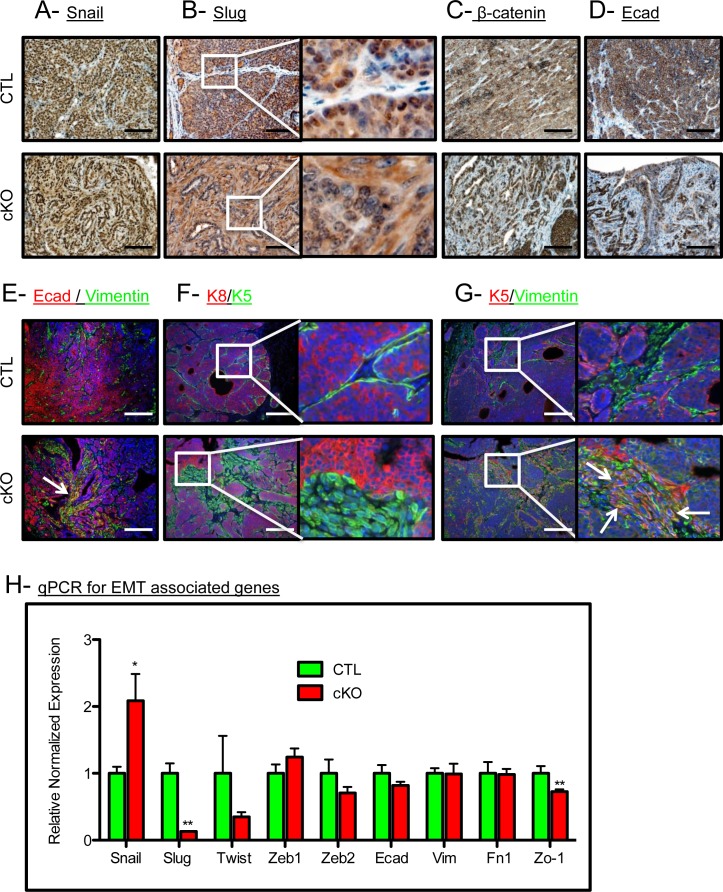
BMPR1a deletion in mammary carcinomas results in EMT-like changes **A.** Representative image of IHC for the EMT associated transcription factor SNAIL. **B.** Representative image of IHC for the EMT associated transcription factor SLUG. **C.** Representative image of IHC for β-catenin in CTL tumors and cKO tumors. **D.** Representative image of IHC for E-cadherin in CTL tumors and in cKO tumors. **E.** Representative image of IF staining for both E-cadherin and Vimentin in CTL tumors and co-staining in cKO tumors. **F.** Representative image of Luminal (K8) and basal (K5) cytokeratins in CTL tumors and cKO tumors. **G.** Representative image of K5 and Vimentin in control and cKO tumors (dual staining indicated by white arrows). **H.** qPCR from whole primary tumors for *Snail*, *Slug,* and *Zo-1.* mRNA is normalized to *Gapdh* levels and relative to control tumors. Error bars indicate SEM. *p = < 0.05, **p = < 0.01. Scale bars indicate 200μm.

BMP signaling has been associated with the retention of an epithelial phenotype in tumor cells [30]. Thus, we checked for alterations in epithelial keratin expression and EMT markers in our control and cKO tumors. The EMT associated transcription factor Snail was expressed in the tumor epithelium, while Slug was no longer restricted to the nucleus in the cKO tumors (Figure 2A and 2B). Nuclear localization of β-catenin and the focal loss of E-Cadherin were also associated with an increase in a mesenchymal phenotype in the cKO tumors (Figure 2C and 2D). cKO tumors displayed E-Cadherin localized with Vimentin staining which indicates an EMT-like transition, while the control tumors displayed no co-expression of these markers (co-expression denoted by a white arrow, Figure 2E). Additionally, we found that control tumors had typical expression of K5 (a marker of basal/myoepithelial cells) restricted to the periphery of the epithelium, whereas cKO tumors had K5 positive cells in the stroma surrounding the tumors (Figure 2F). In control tumors, K5 staining remained mutually exclusive of stromal cells positive for Vimentin, yet in the BMPR1a cKO tumors, overlap of epithelial and mesenchymal markers can be clearly seen as denoted by white arrows (Figure 2G). Confirming these histological observations, qPCR analysis showed a higher expression of the EMT inducing transcription factor *Snail* and a decrease in the expression of *Slug* (Figure 2H). This EMT may be driven by TGFβ activation, which is demonstrated in the stroma by phospho-Smad2 staining in BMPR1a cKO tumors (Suppl. Figure 2A). Consistent with the loss of epithelial morphology in BMPR1a cKO tumors was the mis-localization of the basement membrane component Collagen IV. By IHC examination, we see typical restriction of Collagen IV to the surrounding epithelium in control tumors, yet in cKO tumors Collagen IV is expressed on the inside of the tumor mass where epithelial morphology was absent (Suppl. Figure 2B). We next examined the basal marker p63 in combination with K5, and found that these cells typically mark similar populations juxtaposed to the surrounding stroma. Interestingly, BMPR1a cKO tumors again displayed an “inside-out” morphology, whereby the p63 positive cells were no longer at the basement membrane and displayed a “delamination” from the outer epithelium (white arrows, Suppl. Figure 2C). In concordance with the loss of epithelium, loss of differentiation markers of the basal, myoepithelium and luminal lineages was significantly decreased in BMPR1a cKO bulk tumors (Suppl. Figure 2D). While some markers' expression was reduced (such as *p63* in basal cells), *K14* expression remained unaffected. On the more differentiated side of the mammary tumors, there was a significant decrease in luminal marker expression of *Gata3*, yet the levels of K8 and K18 remained unaffected. Only genes exclusive to the myoepithelial lineage are universally decreased, which indicates a loss of K5+ cells and not K14 or K8/18 cells (Suppl. Figure 2D). The data indicate the K5+ cells are undergoing a transition to express mesenchymal markers (Figure 2F and 2G).

### Microarray analyses of tumors reveal unique role of BMP signaling

**Figure 3:**
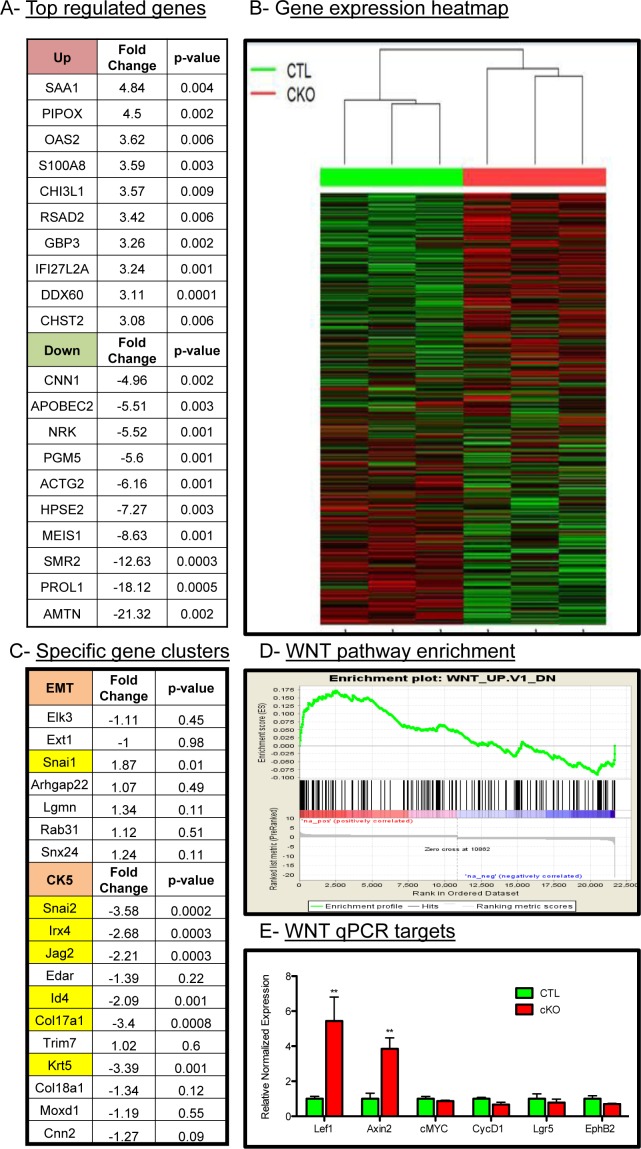
Microarray analysis of control and BMPR1a deleted tumors **A.** Three CTL and three cKO primary whole tumors were analyzed by Affymetrix mouse gene 2.0 DNA microarrays and found to express a set of genes differentially up regulated and down regulated. **B.** Hierarchal clustering of gene expression displayed by heatmap of total gene set by tumor genotype. **C.** cKO tumors contained specific changes in the EMT gene cluster and the basal/myoepithelial gene cluster CK5 were found to contain significant changes in cKO mice. **D.** Among comparison to GSEA gene signatures WNT1 overexpressing tumors were found to be significantly similar to BMPR1a cKO tumors and significantly enriched gene signatures. **E.** qPCR analysis from whole tumors of WNT canonical target genes. mRNA is normalized to *Gapdh* levels and relative to control tumors and fold changes are given in log2 scale. Error bars indicate SEM. **p = < 0.01.

Given the histological changes in the tumors upon loss of BMPR1a, we sought to delineate the mechanisms of action for these phenotypes by large-scale gene expression analysis. We performed Affymetrix gene mouse microarrays from RNA of whole tumors, and found a set of genes significantly upregulated and downregulated in BMPR1a cKO tumors relative to control tumors (Figure 3A). These targets were validated through quantitative PCR analysis (Suppl. Figure 3A); a complete list of gene expression changes is included in Suppl. Table 2. The gene signatures of the BMPR1a cKO tumors showed that these tumors cluster with each other and were distinct from control tumors (Figure 3B). Consistent with the histological evidence for the promotion of an EMT-like transition, microarray analysis showed an up-regulation of the EMT associated transcription factor *Snai1* and a loss of differentiation markers such as *Id4* (also a known BMP transcriptional target) and *Krt5* as well as *Snai2* (also known as *Slug)* (Figure 3C). We observed a loss of CK5 cluster gene expression and not the CK14 cluster, which are distinct populations in mouse tumor models [31], which is consistent with our qPCR analysis previously shown (Suppl. Figure 2D). These results indicate a role for the CK5 or myoepithelial gene cluster via BMPR1a signaling (Figure 3C). Gene signature analysis showed that there was a significant enrichment of WNT signaling targets in the BMPR1a knockout model (Figure 3D). This finding in our microarray data was validated through qPCR analysis of several identified Wnt targets (Figure 3E). We next took our gene expression data and compared our mouse orthologues to the human genes used in the PAM50 gene expression set to classify human tumors [32]. We found no significant changes in the genes that represent the five molecular types of human breast cancer within our cKO tumors compared to our control tumors. However, the five human molecular subtypes do not delineate basal genes from myoepithelial genes as do mouse gene clusters (Suppl. Figure 3C).

### BMPR1a null primary tumor cell lines have decreased tumorigenecity

**Figure 4:**
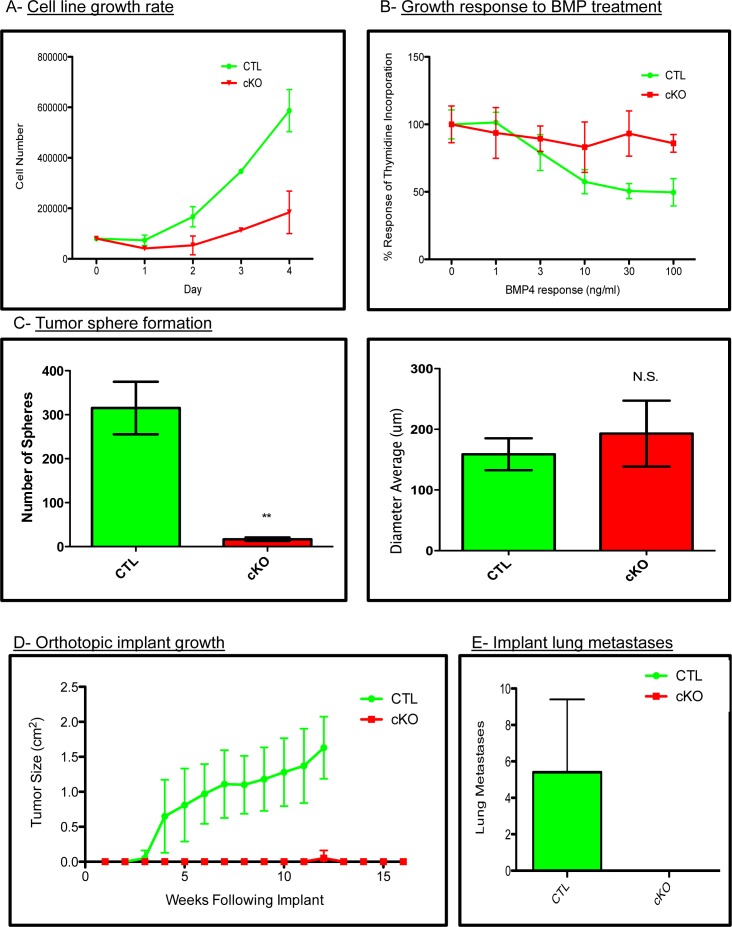
BMPR1a null primary tumor cell lines have decreased tumorigenicity **A.** Total cell counts indicating proliferation rates of control and cKO tumor cells. **B.** Proliferation of CTL and cKO tumor cells in response to BMP (100ng/mL) as determined by tritiated thymidine incorporation after 48 hours of ligand stimulation. **C.** Generation of tumor spheres from three control and three cKO primary cells after 10 days in non-adherent culture conditions. **D.** Syngeneic cells were orthotopically implanted into the #4 mammary gland of C57BL6 females in collagen plugs and allowed to form tumors. **E.** Gross lung metastatic burden as determined by lung whole mounts in CTL and cKO tumor bearing mice. Scale bars indicate 200μm. Error bars indicate SEM. **p = < 0.01.

To test whether our *in vivo* phenotypes were due to cell intrinsic properties or potentially microenvironmental effects, we established primary cell lines from the BMPR1a conditional knockout model. All cell lines were genotyped to verify the expression of the PyMT oncogene, Wap.Cre, mTom/mGFP Cre reporter, floxed BMPR1a alleles and the deletion of exon2 from the BMPR1a gene (Suppl. Figure 4A). We next tested cells for their ability to respond to BMP signaling by measuring induction of the canonical BMP transcriptional targets *Id1*, *Smad6* and *Smad7*, which were induced in control tumor cells and not in cKO tumor cell lines (Suppl. Figure 4B). Additionally, BMP treatment of cKO cells was unable to induce phosphorylation of BMP canonical Smads 1/5/8 as seen in the control cells (Suppl. Figure 4C). We found cKO cells to proliferate significantly slower than CTL cells (Figure 4A). Stimulation of breast cancer cells with BMP ligands is known to reduce proliferation [16]. We treated our cells with an increasing concentration of BMP4 and observed decreased proliferation in control tumor cells yet no significant change in our cKO cells by thymidine incorporation (Figure 4B). Equal numbers of control and cKO cells were seeded in non-adherent culture conditions, and we quantified the number of spheres that formed after ten days. Control tumor cells formed significantly more spheres than cKO cells, although the size of the spheres after ten days of growth was similar (Figure 4C). Next, we examined the ability of our cells to form tumors upon orthotopic injection. Cells were of pure C57BL6 background, which allowed for orthotopic implantation into #4 mammary glands (*n* = 5 for each group). Control tumor cells formed palpable tumors by 3 weeks post implant and required euthanasia for health reasons by 12 weeks of age (Figure 4D). cKO cells did not form tumors except for one mouse at 12 weeks post implant. Additional cKO cell implantations were performed and mice were allowed to live until 16 weeks. Upon examination, grossly and histologically, there was no evidence of tumors (Figure 4D). To ensure that the cKO cells had not simply metastasized directly to the lungs, we performed wholemount analysis and subsequent sectioning of lungs from implant mice and found evidence of lung metastases only in CTL cell implanted mice (Figure 4E).

### BMPR1a null primary tumor cell lines have delayed wound closure

**Figure 5:**
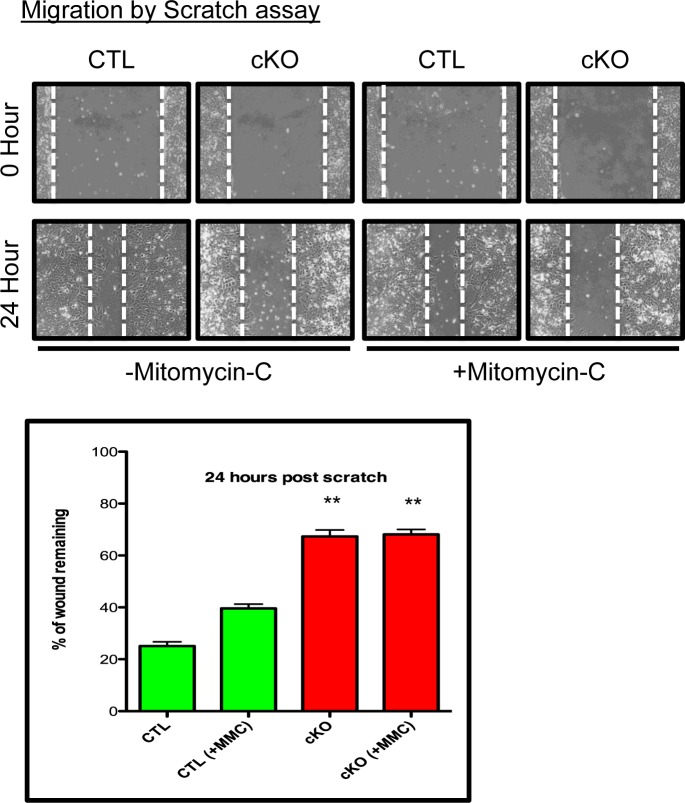
BMPR1a null primary tumor cell lines have delayed wound closure Representative image of scratch wounding assay with CTL and cKO primary tumor cells after confluence with and without proliferation inhibitor Mitomycin-C (MMC). **p = < 0.01.

To further explore the consequences of BMPR1a conditional deletion, we chose to measure the ability of the primary tumor cells to migrate and invade. Using a scratch assay, CTL cells were capable of ‘healing the wound’ to 20% of its original distance, while cKO cells could only ‘heal’ to 60% of the wound distance after 24 hours (Figure 5A). Because proliferation contributes to a scratch assays closure and cKO cells grow at a slower rate, we eliminated contributions from proliferating cells by blocking proliferation with Mitomycin-C (MMC) treatment. MMC did reduce, as expected, the ability of CTL cells to close the wound distance, yet there was no change in cKO cells, and they remained significantly impaired in their ability to close the scratch in comparison to CTL cells (Figure 5). We additionally examined invasion of primary tumor cell lines through Matrigel, which showed high variability amongst the cell lines, but no significant difference between the control and cKO cells (data not shown).

### BMPR1a expression correlates with tumor aggressiveness in human breast cancer

**Figure 6:**
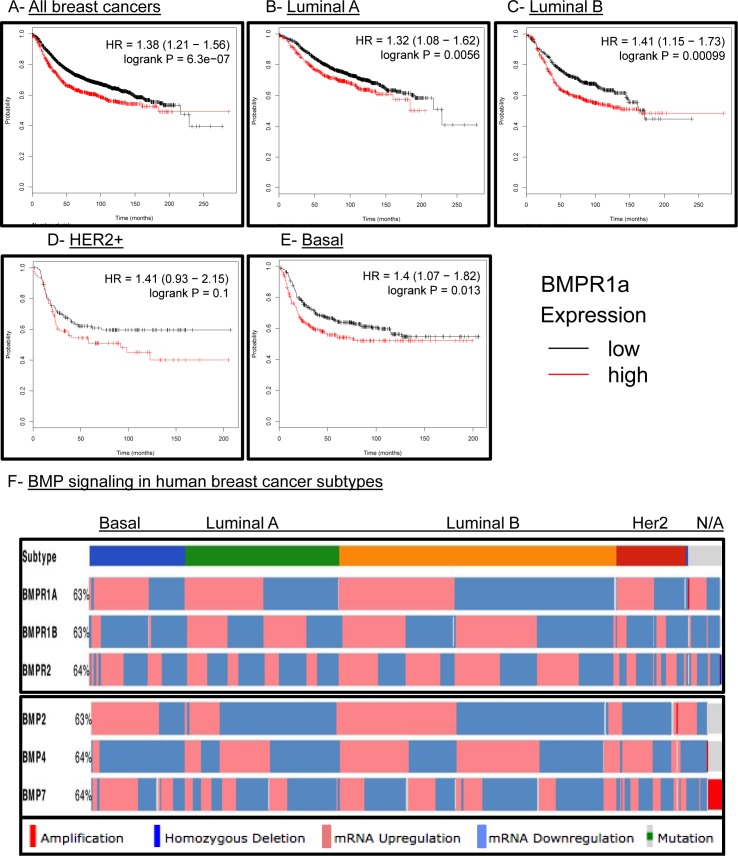
BMPR1a correlates as a tumor promoter in human breast cancer and is not unique to any molecular subtype **A.**-**E.** kmplot.com breast cancer survival analysis of *BMPR1a* expression in All, Luminal **A.** Luminal **B.** HER2+ and Basal breast cancers. Red lines indicate high *BMPR1a* expression and black lines indicate low expression. **D.** TCGA data viewed by the cBio.org portal with expression of BMP receptors and BMP ligands segregated by molecular subtype of breast cancer. Percentages indicate percent alteration or mutation of each gene in relation to all breast cancer tumors available in this database.

We had previously observed that human breast cancers had strong staining for active BMP canonical Smads 1/5/9 [33]. We had also reported that high BMPR1a expression was the only type I TGFβ receptor to correlate with poor relapse free survival (RFS) in human breast cancers [33]. Because we did not observe an association of our BMPR1a cKO tumors with a molecular subtype of breast cancers (Suppl. Figure 3C), we hoped to determine whether BMPR1a expression correlated with changes in RFS in all breast cancer subtypes. We observed that high expression of BMPR1a is strongly associated with poor RFS (Figure 6A). When broken down into specific molecular subtypes such as Luminal A (Figure 6B), Luminal B (Figure 6C), HER2 amplified (Figure 6D), Basal breast cancers (Figure 6E), or estrogen receptor positive and negative (Suppl. Figure 5A), we found that all of these molecular subtypes showed a correlation of high BMPR1a expression with poor RFS. Interestingly, in the case of progesterone receptor (PR) status as well as lymph node spread, no statistical significance was determined for high or low BMPR1a expression and RFS (Suppl. Figure 5B and 5C).

The Cancer Genome Atlas (TCGA) has recently provided cancer researchers with tools to compare large human data sets [34]. When queried for the three most common BMP receptors: BMPR1a, BMPR2 and BMPR1b, BMPR1a and BMPR2 are largely equally distributed for high and low expression between the four largest molecular subtypes of breast cancer. Only BMPR1b shows a bias to be downregulated in basal type breast cancers and no other subtype (Figure 6F). When queried for the three most common BMP ligands: BMP2, 4 and 7, we observe that basal cancers have upregulation of BMP2 and BMP7 and downregulation of BMP4 (Figure 6F). Interestingly, BMP2 while upregulated in basal breast cancers is more frequently downregulated in Luminal A and HER2 amplified subtypes (Figure 6F). We also investigated other components of the BMP pathway such as the ligand antagonists *DAND5* (also known as *COCO*), Chordin, Chordin-like 2, and Gremlin 2. We found as previously observed that DAND5 was more commonly upregulated in basal cancers [15] and downregulated in Her2 amplified cancers. Basal cancers also showed that Chordin-like 2 (*CHRDL2*) was also upregulated while Chordin (*CHRD*) and Gremlin 2 (*GREM2*) were downregulated (Suppl. Figure 5D). BMP signaling is canonically mediated by Smads, which can be shared by other TGFβ family signaling molecules. BMP specific Smads 1,5,6 and 9 did not show a significant distribution of expression changes between molecular subtypes of human breast cancer (Suppl. Figure 5D). Overall, these results indicate that BMP signaling may contain unique subtype changes only with select receptor-ligand-antagonist compositions.

## DISCUSSION

When BMPR1a was first identified as the gene responsible for the formation of polyps in JPS patients, it was quickly labeled a tumor suppressor, because loss of normal functioning BMPR1a results in polyp formation [6]. Yet even after this discovery, it was clear that these polyps were unlike many of the aggressive adenomatous intestinal polyps caused by WNT activation, and it was observed that BMPR1a polyps would only rarely progress to carcinoma [35]. Another interesting observation about JPS is the general lack of epithelial cells within the polyp itself that histologically separates it from other intestinal polyps and cancers. When several groups conditionally deleted BMPR1a in the skin, they all found that spontaneous hair follicle like hamartomas were formed, but not squamous cell carcinomas or basal cell carcinomas[7-10]. Our results indicate that when cancer is already formed and driven by a powerful oncogene that is capable of metastasis, the loss of BMPR1a reduces tumor burden and metastatic potential.

We suspect that this unique morphology found focally in our mouse model and in JPS patients is derived from changes due to EMT. Many studies have found that BMP signaling antagonizes the TGFβ directed EMT program, and that loss of BMP signaling results in WNT and TGFβ cooperatively driving the EMT process [30]. Concordantly, it has been shown in breast and prostate cancer that tumors use TGFβ to drive EMT to escape the primary tumor site and then reverse the process with BMP signaling driving the Mesenchymal-to-Epithelial-Transition (MET) to colonize the distant metastatic site [36]. We find significant differences in EMT appearance by protein via IF staining when compared to RNA expression, likely indicative of the dynamic nature of EMT structural components. While TGFβ is the best studied inducer of EMT, there are other factors that regulate the EMT and MET switch in concert or independently of TGFβ. TGFβRII cKO tumors have accelerated tumor growth and metastasis, yet lack the ability to signal canonically through TGFβ, therefore it remains unknown what pathways are driving the EMT and MET transitions resulting in metastatic colonization [37]. In this study we have increased EMT upon loss of BMP signaling, yet these cKO tumor cells do not form tumors or metastases (Figure 4D and 4E). The lung metastases that are formed (in the spontaneous tumors) are from un-recombined cells, which indicate a preference for BMP signaling in the establishment of overt metastatic lesions in the lung. Interestingly, recent work from Drasin et al. supports the induction of a sustained EMT being detrimental to the formation of metastatic lesions[38]. Additionally, the isolation of purely mesenchymal tumor cells from a model of prostate cancer shows that these Vimentin positive cells showed reduced capacity to form metastatic lesions upon tail vein injection when compared with their Vimentin negative counterparts[39]. While the lack of migration in our EMT BMPR1a knockout cells might seem counterintuitive, we believe that BMP signaling acts in concert with other signaling pathways to illicit tumor cell migration independently. This tumor cell migration ends with loss of BMPR1a expression[40]. This work is supported by our metastatic data showing metastatic lesions in our spontaneous mouse model having active BMP signaling, as well as the inability of primary tumor cells to form tumors upon orthotopic implantation.

BMPs have generally been thought of as tumor suppressors; however, they have been shown to have tumor promoting roles in cancer as well. This finding is similar to that of the dual roles of TGFβ in cancer formation and progression. Recent reviews have highlighted the tumor suppressive and newly discovered tumor-promoting roles of BMP signaling [25, 26]. That BMP signaling can drive tumor progression has been the impetus behind the use of pharmacologic small inhibitors to the type I BMP receptors, such as BMPR1a, to inhibit BMP signaling. One such compound, LDN-193189, has been used successfully to limit tumor growth in breast, prostate and lung cancer mouse models. Treatment of a mouse model of breast cancer resulted in the reduction of ALDH1+ cancer stem cells. In a prostate cancer model, pharmacologic inhibition of BMP signaling reduced the colonization of the bone [19-21, 41], which has also been shown genetically with loss of BMP receptors [21]. Our recently published work demonstrates that BMP inhibition not only has an effect on tumor cells but also on the surrounding microenvironment, which may also work in concert via BMP signaling to coordinate tumor progression [33].

Human breast cancers are well known to be heterogeneous, both from patient to patient, within the primary tumor and over the course of progression, treatment and relapse. It is a long-standing fundamental question in cancer to determine the most aggressive cancers from the indolent ones. BMP signaling may represent a pathway in cancer that can separate cancers into treatment options. Recently it was shown that deletion of BMPR1a in colon cancer can sensitize cells to chemotherapy, and that the constitutively active form of BMPR1a can drive chemo-resistance [24]. Our data suggest that BMPR1a has a function unrelated to breast cancer molecular subtypes, which suggests that targeting BMPR1a is a potential therapy for all subtypes of breast cancer. These studies indicate that BMP receptors have distinct tumor promoting and suppressive mechanisms that warrant further studies in distinct cancer-related contexts. Such data implies that it is not only the ability of a cell to undergo EMT which is important, but also the reverting to an epithelial phenotype in metastatic spread. These discoveries could be an important consideration for patient prognosis and treatment.

## MATERIALS AND METHODS

### Animals

All animal experiments were performed at Vanderbilt University and approved by IACUC (Internal protocol #M/04/192). All animals were used within the standards as prescribed by “Guidelines for the welfare and use of animals in cancer research” (Workman et al., 2010). All animals were used in the congenic background C57BL6 by backcrossing and validation from animals and genetic testing services from Charles River Laboratories (Ithaca NY). MMTV.PyMT [27] mice were bred with Wap.Cre [28] and BMPR1a floxed [29] mice and genotyped as describe in the original publications. For cell line development and Cre validation Cre reporter mice were purchased and bred to the above combination of mice. These mice express tdTomato in all cells and under the control of Cre switch from tdTomato to express mGFP [42].

### Histology and lung whole mount procedures

Lungs were fixed in 10% neutral-buffered formalin overnight at 4°C. The next day, lungs were dehydrated, placed in xylene for 1 hour, and then changed to fresh xylene overnight. Lungs were rehydrated before dipping in Mayer hematoxylin for 2 minutes and then washed in running tap water for 5 minutes. Tissues were destained in HCl (fresh 1% v/v from a 12 N solution) for 20 minutes, rinsed in running tap water overnight, dehydrated, and placed in xylene overnight before counting stained metastatic tumor foci under a dissecting light microscope. After whole mount analysis, lungs were paraffin embedded and processed for H&E sections and normal histological analysis.

### Immunohistochemistry and immunofluorescence

Paraffin tissues were embedded and sectioned at 5μM and dewaxed in xylene and rehydrated in alcohol with citrate antigen retrieval as previously described [5]. Standard Mayer's hematoxylin and eosin (H&E) was performed. Cleaved Caspase-3 (Cell Signaling Cat#9661, 1:200), Vimentin (Covance Cat#PCK-594P 1:500), BrdU (BD Cat#563445 1:100). pSmad1/5 (Cell Signaling Cat#9516 1:200 ), Snail (Santa Cruz Cat#28199 1:200), Slug (Santa Cruz Cat#166476 1:100), Ecadherin (BD Cat#610181 1:200), K8/18 (Fitzgerald Cat#20R-CP004 1:500), K5 (Covance Cat#PRB160P 1:500), pSmad2 (Cell Signaling Cat#3101 1:500), CollagenIV (Abcam Cat#19808 1:500) and p63 (Santa Cruz Cat#8344 1:200). Paraffin derived sections were counterstained with hematoxylin (Vector Labs QS) and mounted with Cytoseal. Immunofluorescence staining was performed with primary and secondary antibodies diluted in 12% Fraction-V BSA (Pierce) and slides were mounted in SlowFade mounting medium containing DAPI (Invitrogen). All fluorescent secondary antibodies were highly cross-adsorbed, produced in goat and used at a dilution of 1:200 for 20 min (Molecular Probes). Quantification of IHC and IF was performed using NIH ImageJ (http://rsbweb.nih.gov/ij/docs/examples/stained-sections/index.html) as previously described [43].

### RNA isolation, cDNA synthesis, qPCR and primer selection

RNA isolation was performed by placing tissue directly into Trizol (Invitrogen) and purified by chloroform and alcohol precipitation. RNA was then subjected to cleanup with RNeasy purification including DNAseI treatment (Qiagen). Equal amounts of RNA were synthesized into cDNA using the VILO cDNA synthesis kit (Invitrogen). LuminoCt (Sigma) 2X SYBR master mix was combined with 1 μM of both a forward and reverse primer sequence (full table of sequences is listed in Suppl. Table 1) into 20 μl reactions and cycled for 95°-10 s to 60° for 30 s for 40 cycles followed by a melting curve. BioRad CFX96 was used and instrument provided software was used to determine relative normalized expression relative to *Gapdh* expression.

### Microarray, analysis and gene signature comparisons

Microarray data were processed and quality controlled using Affymetrix's Expression Console software. Differential analysis were conducted using LIMMA package [44] in R. Heatmaps and cluster analyses on the combined data were generated using heatmap 3 package [45] in R. Functional analysis was conducted using Gene Set Enrichment Analysis (GSEA) [46].

### Cell culture

Primary tumor cell lines were established by digesting primary tumors with Dispase, Collagenase 3, DNase and antibiotics (Worthignton Bio) for two hours in 37-degree shaker water bath. The following digestion was filtered with a 40μm cell strainer and reversed washed and plated into a T-75 tissue culture flask containing DMEM/F12 medium with 5% Adult Bovine Serum (ABS) and triple antibiotic/antimycotic (Life Technologies). Cells were allowed to grow to confluence and form domes. They were inspected for GFP/Cre expression and after passaging through growth crisis were sorted using flow cytometry. Routine mycoplasma testing was performed and cells were treated for 24 hours prophylactically with ciprofloxin for 24 hours after cell sorting (GeneHunter).

### Western blot

Total protein was isolated using Complete LysisM Buffer (Roche). Protein was diluted to equal concentrations and equally loaded on 10% polyacrylamide gels prior to transfer to a nitrocellulose membrane. Protein concentration was determined using micro plate BCA assay (BioRad). Blots were incubated overnight with PyMT (Santa Cruz Cat#53481 1:1000), pSmad1/5 (Cell Signaling Cat#9516 1:1000), and Actin (Sigma Cat#A2066 1:4000) antibodies. HRP-conjugated secondary antibodies were used to visualize band intensity via x-ray film exposure using ECL western substrate (Perkin Elmer).

### Scratch migration assay

Cell migration was assessed using a standard monolayer scratch assay (Russell et al., 2003). Cells were removed from the center of a confluent monolayer of cells with a p200 pipette tip and the growth media was changed to remove floating cells from the dish. The width of the scratch was imaged and measured (at ∼ 50 areas per dish) before and after incubation (0 and 18 hours; 37 °C) at 10X magnification. Percent wound closure was calculated as follows: 100 minus average final wound width)/average initial wound width X 100 [43, 47].

### Human breast cancer database analysis

For analysis of the TCGA data set, we used the cBio portal (http://www.cbio.portal.org/) [34, 48]. Human gene symbols were queried in the provisional data set and accessed on 11 February 2015. RNA expression cutoff was adjusted to 0.01 to determine total cutoff high or low expression in samples from the median. Analysis of gene expression correlating with relapse free survival (RFS) was performed using the kmplotter (http://kmplot.com). Human gene symbols were entered into breast, and JetSet probe selection was used to determine optimal representative microarray probe [49]. Automatic cutoff scores were selected during queries and 10-year RFS were selected. Statistical analysis was performed using Excel (Microsoft, Redmond, WA, USA), Prism (Graphpad, La Jolla, CA, USA), and FlowJo (TreeStar, Ashland, OR, USA) software. Statistical significance for any comparison was *P* < 0.05.

## SUPPLEMENTARY MATERIAL FIGURES AND TABLES






