# Supplementary material for: Deletion of the BMP receptor BMPR1a impairs mammary tumor formation and metastasis
Source: Oncotarget. 2015 Jun 10;6(26):22890–904. doi: 10.18632/oncotarget.4413 (PMC4673207; doi:10.18632/oncotarget.4413)
Supplement: Supplementary file 1 [file oncotarget-06-22890-s001.pdf]

Deletion of the BMP receptor BMPR1a impairs mammary tumor formation and metastasis

Supplementary Material

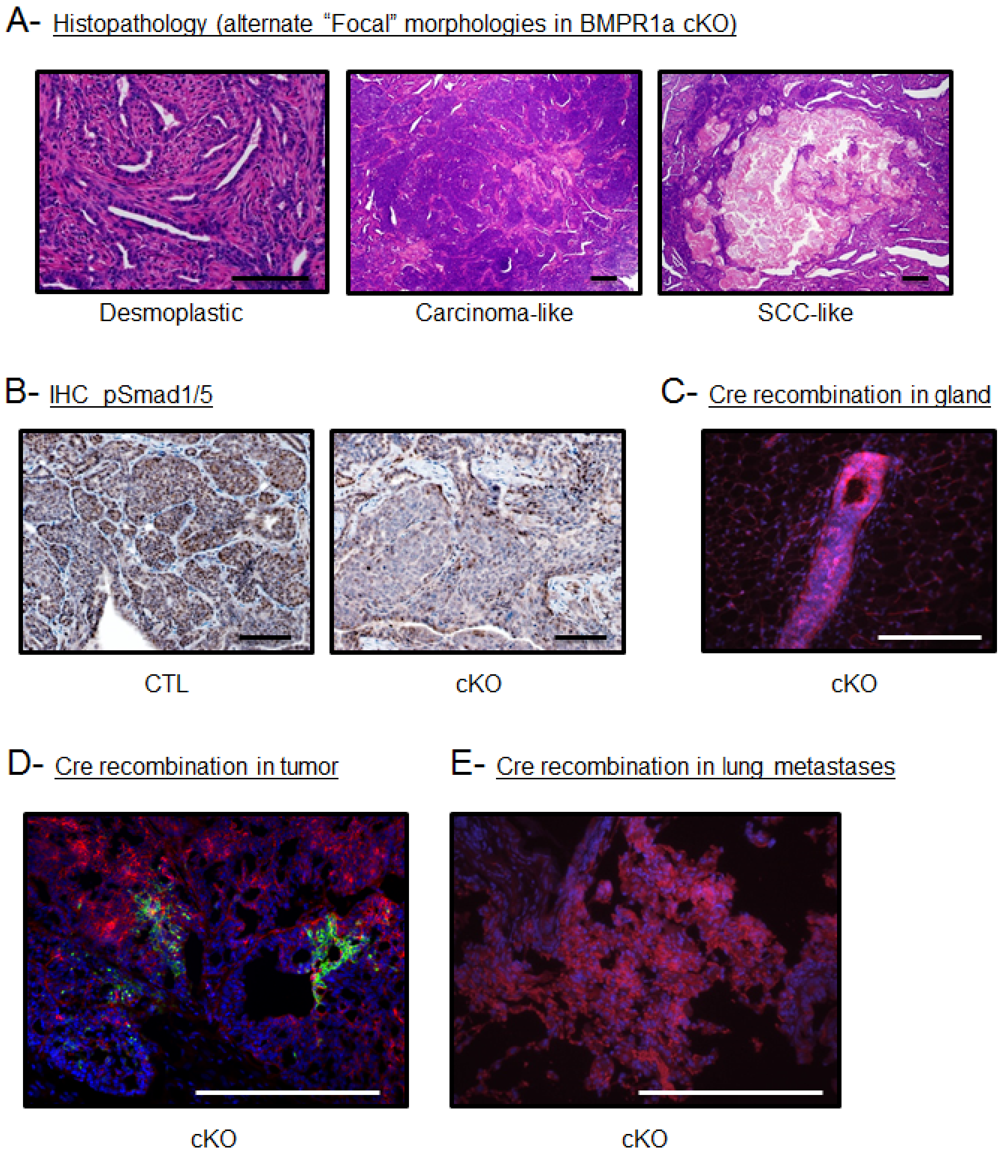

Supplemental Figure 1: Histological analysis of BMPR1a cKO tumors. A. Representative image of H&E staining of cKO tumors demonstrates alternate focal morphologies present in tumors, which vary from desmoplastic, advanced carcinoma and squamous cell carcinoma (SCC)-like. B. Representative image of IHC for BMP canonical response phospho-Smad 1/5 in CTL tumors and cKO tumors. C. Cre recombination was measured in virgin glands from Wap.Cre+ cKO glands prior to tumor formation. D. Cre recombination detected by mGFP (green staining) expression in primary tumors. E. Cre recombination in cKO lung metastases (green if knockout Cre expression and red if no recombination). Scale bars indicate 200µm.

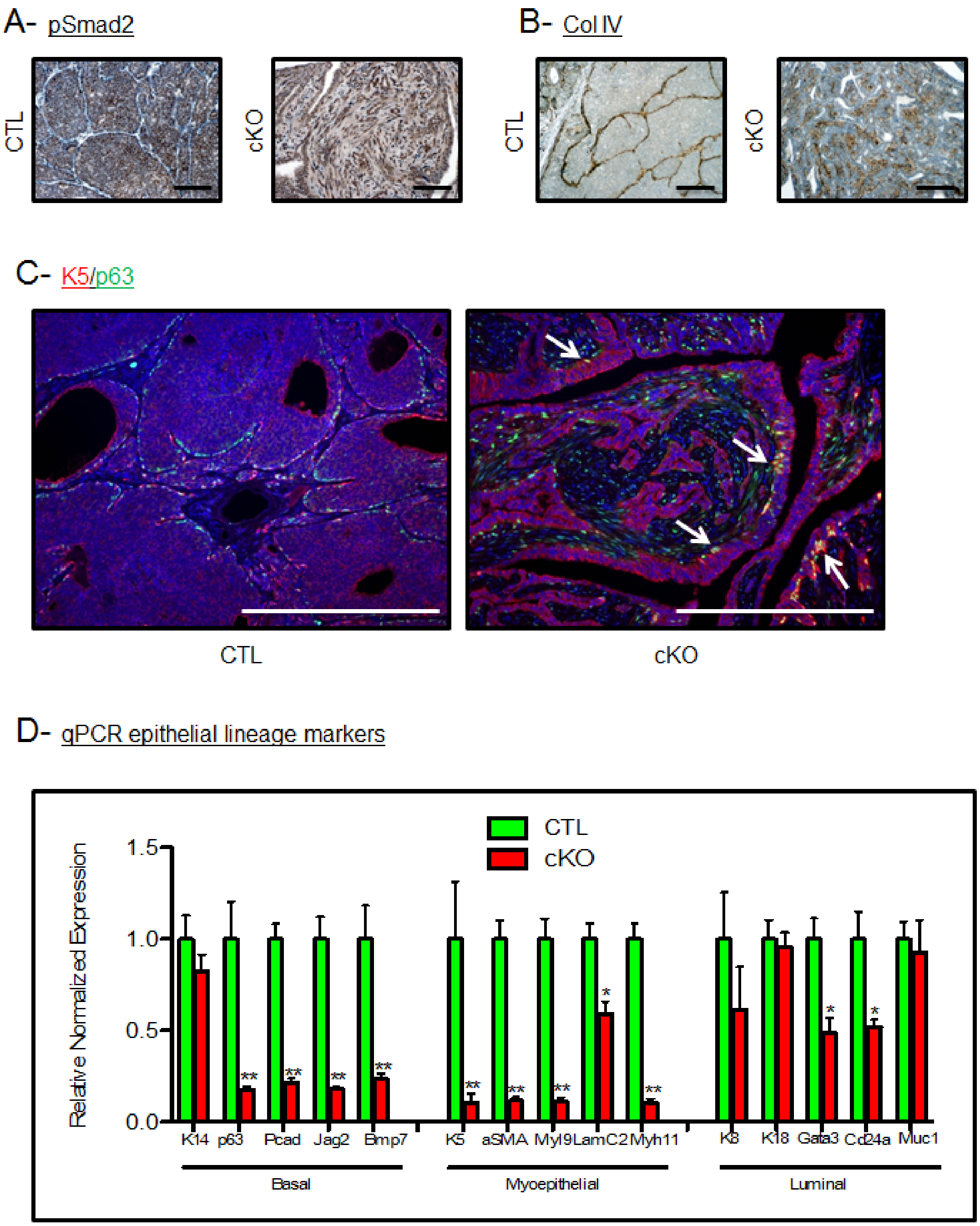

Supplemental Figure 2; BMPR1a cKO developmental lineage defects. A. Representative image of IHC for pSmad2 of both CTL and cKO primary tumors. B. Representative image of IHC for Collagen IV in CTL tumors yet dispersed within the cKO tumors. C. Representative image of IF Staining for K5 and p63 (white arrow indicates delaminating p63+ cells). D. qPCR for lineage markers. mRNA is normalized to *Gapdh* levels and relative to control tumors and fold changes are given in log2 scale. Error bars indicate SEM. *p=<0.05, **p=<0.01. Scale bars indicate 200μm.

A- qPCR Validation of microarray

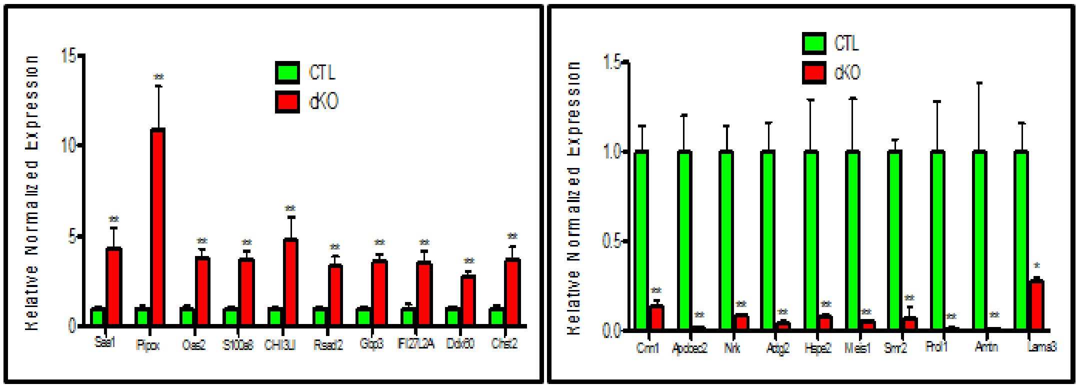

B- Molecular Subtyping of mouse BMPR1a cKO tumors

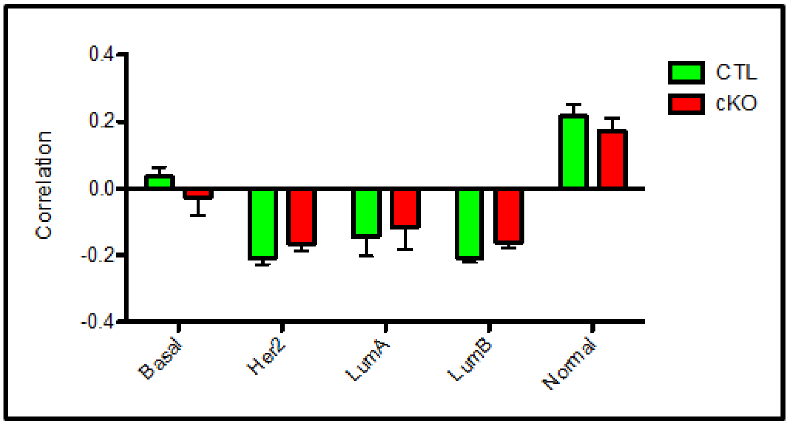

Supplemental Figure 3: Validation of microarray gene signature A. qPCR validation of top 10 up regulated and down regulated genes. B. Correlation to human molecular subtyping using the PAM50 gene set did not display any significant changes in cKO tumors (3 vs. 3 from microarray values). mRNA is normalized to *Gapdh* levels and relative to control tumors. Error bars indicate SEM. *p=<0.05, **p=<0.01.

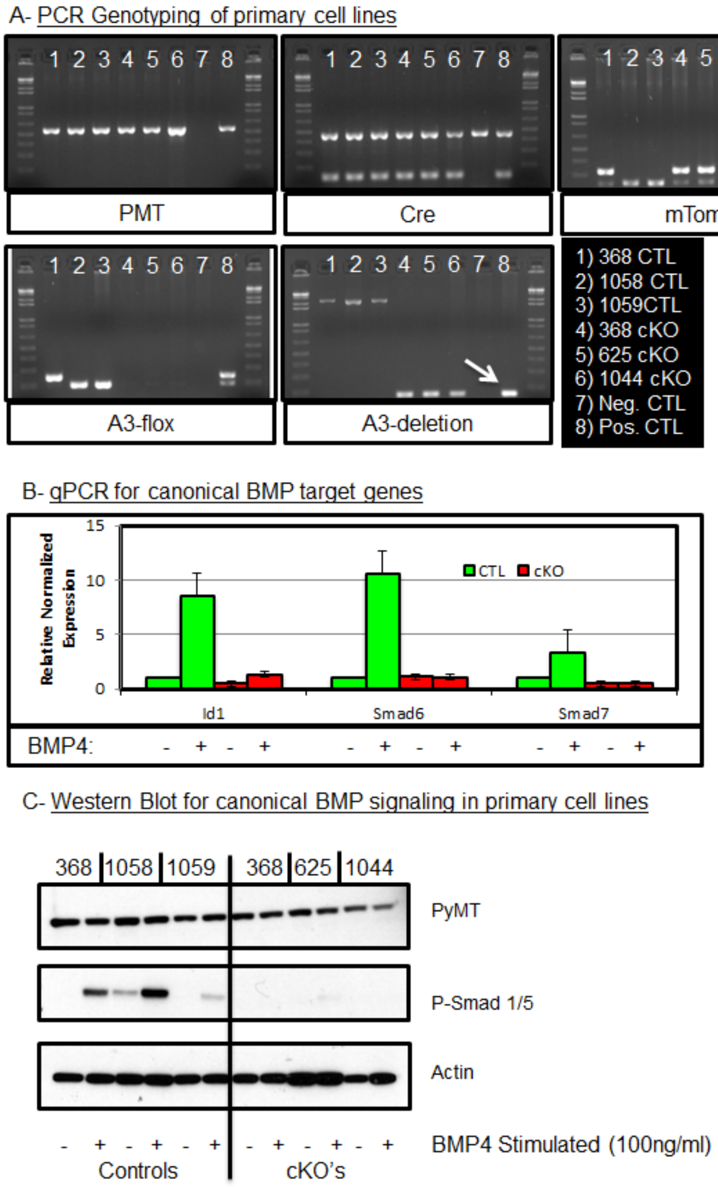

Supplemental Figure 4: Validation of BMPR1a cKO cell lines absence of BMP signaling and failure to transmit canonical signaling. A. PCR genotyping of primary cell lines generated confirmed expression of the MMTV.PyMT oncogene in all cells, Cre expression in all cell lines, at least one copy of the Cre reporter mTom/mGFP allele, BMPR1a (A3) floxed alleles still present in control cells and absent in cKO cells. A unique PCR product generated from recombination was detected in cKO cells and the intact unrecombined DNA was detected in CTL cells (White arrow indicates recombination specific product). B. qPCR for canonical BMP transcriptional targets was exclusively induced in CTL cells. C. Western blots in cell lines validated equal expression of the PyMT oncogene and only induction of phosphorylation of canonical BMP Smads 1 and 5 in CTL cells stimulated with 100ng/ml of recombinant BMP4 for 24 hours. mRNA is normalized to *Gapdh* levels and relative to control tumors. Error bars indicate SEM.

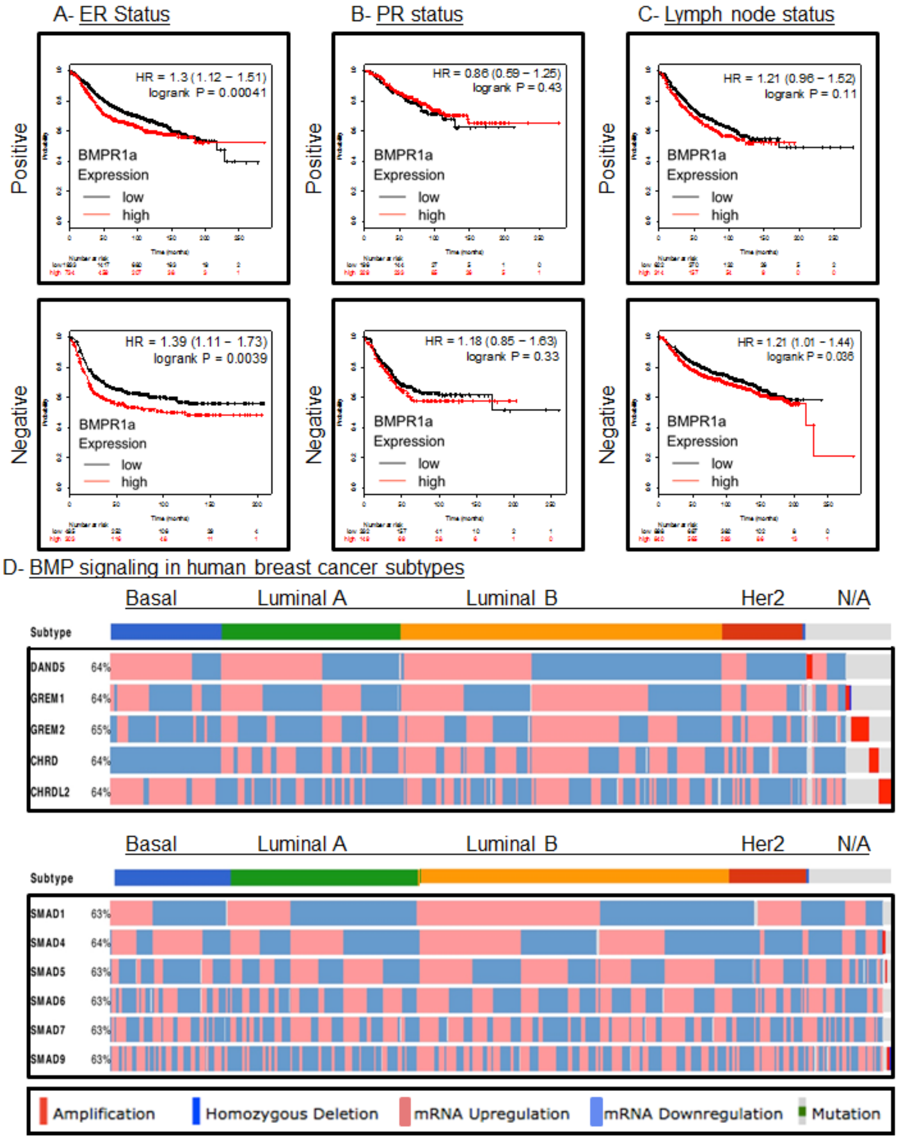

Supplemental Figure 5: Survival and subtype analysis of BMPR1a and additional BMP signaling components in human breast cancer. A-C. kmplot.com breast cancer survival analysis of *BMPR1a* expression in ER, PR and Lymph node status. Red lines indicate high *BMPR1a* expression and black lines indicate low expression. D. TCGA data viewed by the cBio.org portal with expression of BMP antagonists (upper box) and intracellular mediators (Smads) segregated by molecular subtype of breast cancer.

Supplemental Table 1: SYBR Primer Sequences. Forward and reverse primer sequences used are listed and either sourced from validated sequences from Harvard primer bank or NCBI Primer tool. All sequences are optimized for 60-degree melting temperature and result in a single product by melting curve analysis.

Supplemental Table 2: Complete microarray list of gene expression. A list of all genes in Affymetrix Mouse Gene 2.0 with log transformed expression values for three CTL and three cKO primary tumors.
